# Clinical approach to a child with hemophagocytic lymphohistiocytosis and bilateral optic nerve head infiltration: A case report and brief literature review

**DOI:** 10.1002/ccr3.7999

**Published:** 2023-09-28

**Authors:** Farid Shekarchian, Mitra Karimi Amir Abadi, Mehrdad Motamed Shariati

**Affiliations:** ^1^ Eye Research Center Mashhad University of Medical Sciences Mashhad Iran

**Keywords:** disc swelling, hemophagocytic lymphohistiocytosis, visual loss

## Abstract

**Key Clinical Message:**

Infiltrative optic neuropathy in hemophagocytic lymphohistiocytosis is rare but could potentially lead to visual loss. Cytomegalovirus (CMV) optic neuritis, drug toxicity, and CNS involvement with increased intracranial pressure (ICP) are differential diagnoses that have to be considered.

**Abstract:**

In this report, we introduced a known case of hemophagocytic lymphohistiocytosis (HLH) with progressive visual loss due to bilateral optic nerve head (ONH) involvement. A 9‐year‐old boy with a history of HLH from 6 months ago was referred to the ophthalmic emergency department with a complaint of painless progressive blurred vision in his right eye. The fundus examination found an optic disc swelling and peripapillary hemorrhage in the right eye. The left fundus examination showed a mild ONH blurred margin. Systemic evaluations including brain and orbital MRI with gadolinium enhancement and CSF analysis showed optic nerve and brain involvement with tumoral cells. Despite systemic chemotherapy with etoposide, the disease had a progressive course so in the last follow‐up visit, fundus examination revealed disc swelling, retinal edema, and epiretinal hemorrhage in both eyes and visual acuity deteriorated to no light perception and counting fingers in the right and left eye, respectively. ONH involvement in HLH is rare but could be sight‐threatening. Differential diagnoses that should be investigated include neoplastic infiltrative optic neuropathy, cytomegalovirus (CMV) optic neuritis, drug toxicity, and CNS involvement with increased intracranial pressure (ICP).

## 1 INTRODUCTION

Hemophagocytic lymphohistiocytosis (HLH) is a potentially lethal immune system dysregulation in children and adults.^1^ Failure to properly inhibit the immune response leads to constant and excessive activity of the cytotoxic T‐cells, natural killer (NK) cells, and macrophages. Inflammatory reactions caused by the intense activity of the cellular immune system and cytokine storm in tissues lead to multiple organ failure in this disease.^2^

HLH is categorized as primary, which is defined as the presence of a predisposing genetic mutation in the immune system, and reactive to an infectious, inflammatory, or malignant trigger.^3^ The diagnosis is challenging as it has no clinical or laboratory pathognomonic features. Fever, organomegaly, liver dysfunction, cytopenias, coagulopathy, hemophagocytosis, and neurologic dysfunction are common manifestations of HLH.^1^

Ocular involvement is relatively rare in HLH. Unilateral panuveitis, Purtscher retinopathy, trabecular meshwork involvement, and choroidal infiltration with secondary extension to the retina and optic nerve head (ONH) have been reported previously.^4^

This report aims to introduce a child with HLH and bilateral ONH infiltration.

## 2 CASE REPORT

**FIGURE 1:**
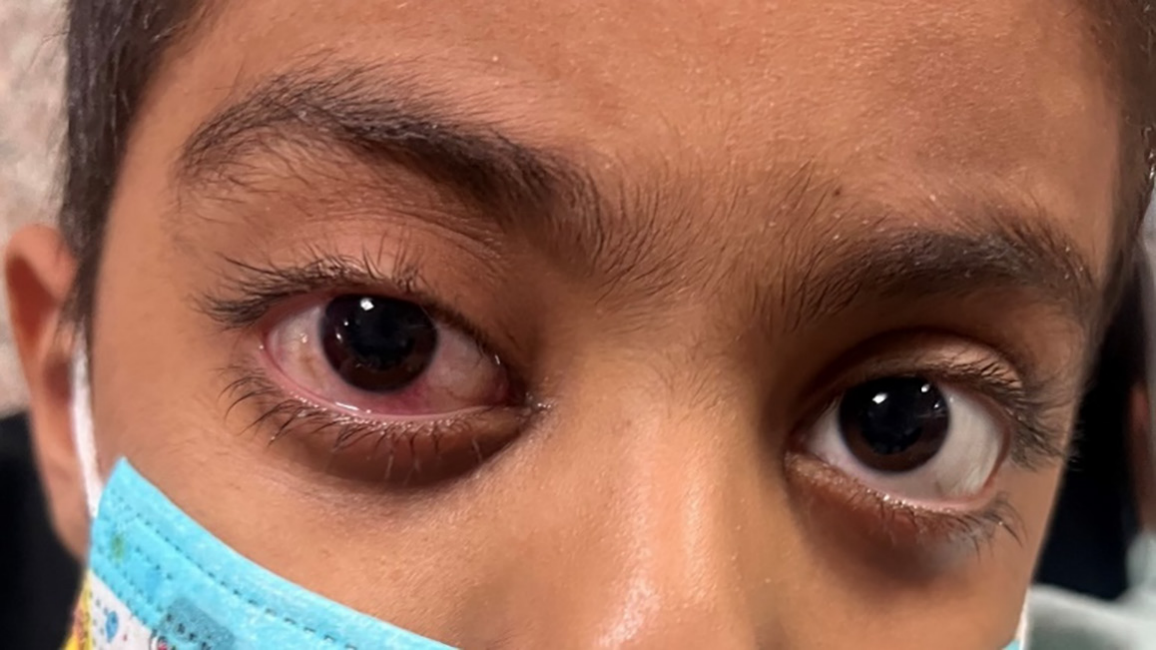
The facial appearance of the patient.

A 9‐year‐old boy, with a history of HLH from January this year, was referred by his pediatric oncologist to the ophthalmic emergency department with a complaint of painless progressive blurred vision in his right eye. On ocular external examination, there was a mild conjunctival injection, chemosis, and proptosis in his right eye (Figure 1). The best‐corrected distance visual acuity was 20/40 and 20/20 for his right and left eye respectively. The relative afferent pupillary defect (RAPD) examination was positive for the right eye. Anterior segment examination was unremarkable for both eyes. In the fundus examination, we found an optic disc swelling and peripapillary hemorrhage in the right eye. The left fundus examination showed a mild ONH blurred margin. Regarding the patient's clinical status, differential diagnoses include neoplastic infiltrative optic neuropathy, cytomegalovirus (CMV) optic neuritis, drug toxicity, and CNS involvement with increased intracranial pressure (ICP). For further evaluation, a brain and orbital MRI with gadolinium enhancement (Figure 3), aqueous sampling with a 25‐gauge needle for polymerase chain reaction (PCR) to detect CMV, lumbar puncture (LP) to analyze cerebrospinal fluid cytology and biochemistry, and also for ICP measurement, and blood test for hematology and biochemistry were ordered. The results of the patient's imaging and lab tests are summarized in Table 1.

**TABLE 1:** laboratory test results.

Laboratory test	Result
Cerebrospinal fluid biochemistry	
Sugar	44 mg/dL
Protein	98.5
Cholesterol	1 mg/dL
LDH	35 U/L
	CSF analysis: mildly increasing lymphomononuclear cells
Complete blood cell analysis and cell differentiation	
white blood cell	5.4
Red blood cell	2.8
Hemoglobin	7.2
Hematocrit	22
MCV	76
MCH	25
MCHC	32.7
Platelet	89
PMN	75
Lymphocyte	25
Glucose	115 mg/dL
Urea	29 mg/dL
Creatinine	0.4 mg/dL
AST	32 U/L
ALT	40 U/L
Calcium	9.3 mg/dL
Sodium	141 meq/L
Potassium	3.8
CRP	5 mgr/dl
ESR	55 MM

**FIGURE 2:**
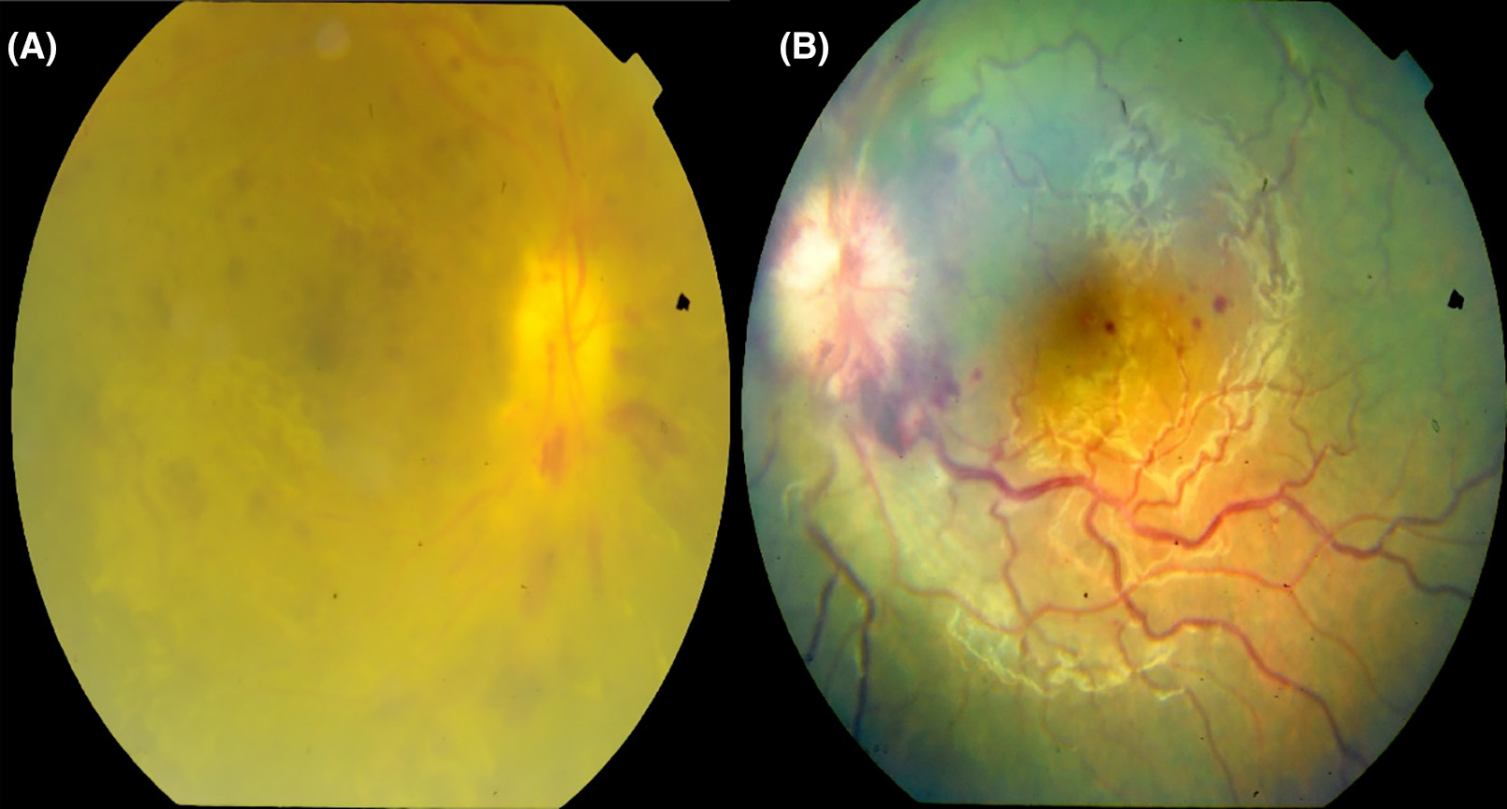
Fundus photograph of the patient. (A) Right eye optic disc swelling and epiretinal hemorrhage. (B) left eye optic disc swelling and splinter hemorrhage around the disc and macular epiretinal hemorrhage.

In the first week of July, in the first follow‐up examination, the optic disc swelling progressed (Figure 2) in both eyes and the visual acuity decreased dramatically to light perception in the right eye and 20/40 in the left eye. The patient was referred to his oncologist to continue systemic chemotherapy and radiotherapy. In the last follow‐up visit, the fundus examination revealed disc swelling, retinal edema and epiretinal hemorrhage in both eyes, and visual acuity deteriorated to no light perception and counting fingers in the right and left eye, respectively.

**FIGURE 3:**
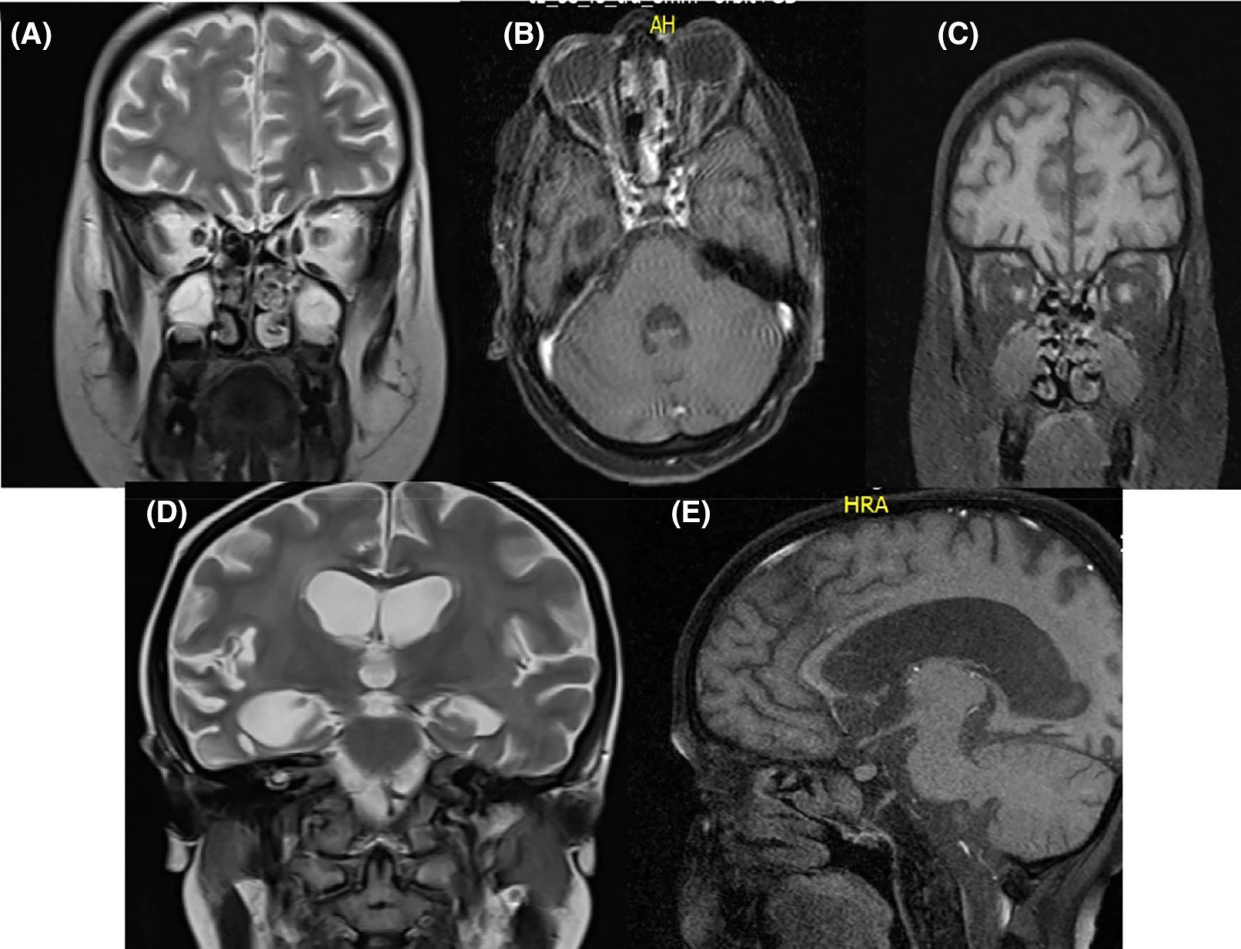
Brain and orbital MRI with and without gadolinium contrast. (A) coronal T2 MRI without contrast shows bilateral optic nerve enlargement (B) axial T1 MRI with contrast shows bilateral optic nerve enlargement and enhancement (C) coronal T1 MRI with contrast shows bilateral optic nerve enlargement and enhancement (D) coronal T2 MRI without contrast shows brain third and lateral ventricles enlargement (E) sagittal T1 MRI with contrast shows brain third ventricle enlargement.

**FIGURE 4:**
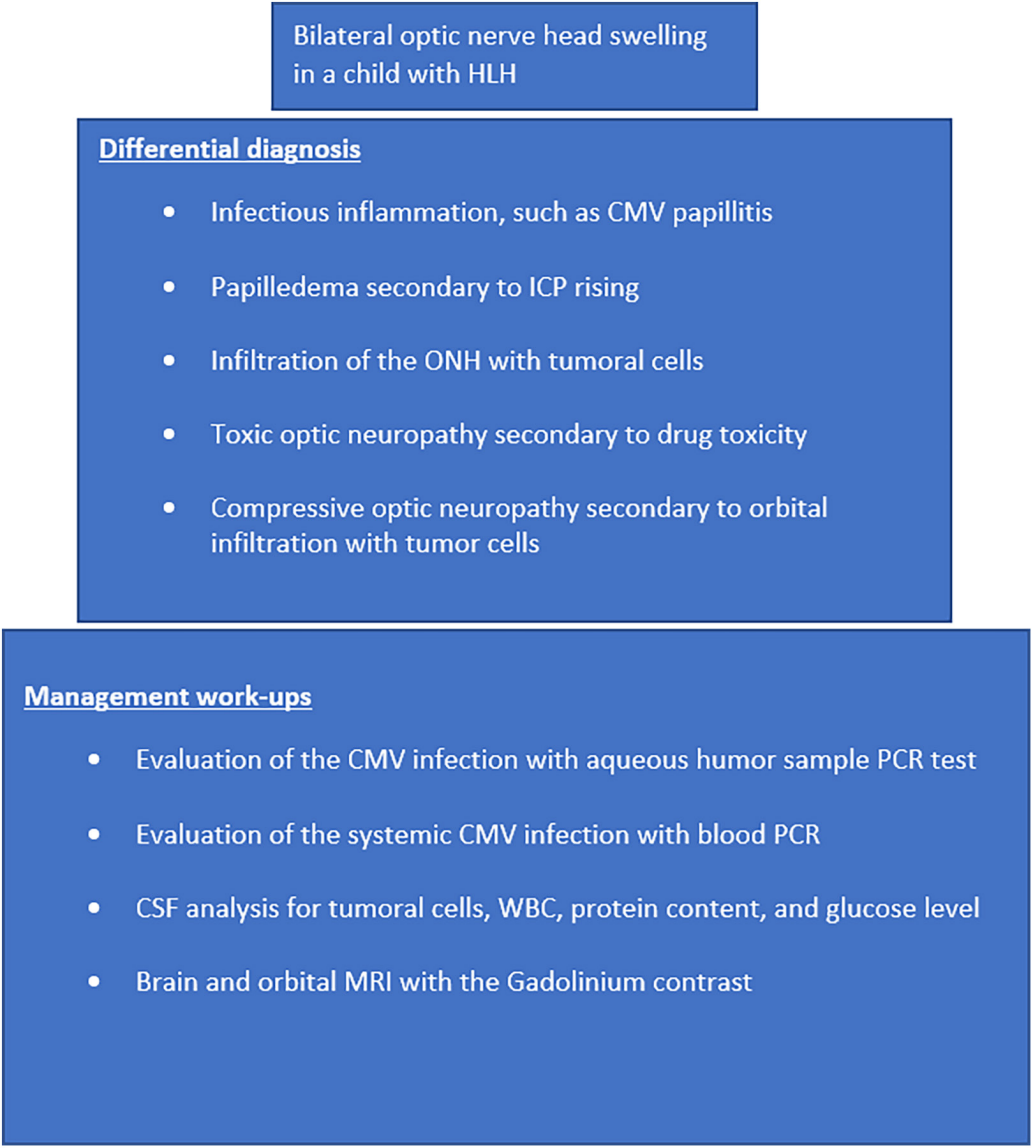
Approach diagram to a patient with HLH and bilateral optic nerve head swelling.

## 3 DISCUSSION

In this study, we reported a child with HLH presented to us with bilateral painless progressive visual loss. In the ophthalmic examination, we found bilateral optic disc swelling and hemorrhage. HLH is an immune system dysregulation characterized by multiorgan infiltration of macrophages, especially affecting the liver, spleen, lungs, bone marrow, lymph nodes, and kidneys leading to various signs and symptoms. The mean age of occurrence is 1.8 in children and 50 years old in adults.^3^

Ocular involvement is uncommon in HLH. Wang et al., in a study in 2023, showed that old age, autoimmune disorders, and decreasing RBC and platelet counts are independent risk factors of ocular involvement in HLH.^4^ Retinal hemorrhage, conjunctivitis, corneal infiltration, anterior uveitis, papillitis, and choroidal involvement are the main ophthalmic signs in these patients.^4^ A brief literature review from 2005 to 2023, including five case reports, one case series, and a retrospective analysis, regarding ocular involvement in HLH is summarized in Table 2.

**TABLE 2:** Summary of previous studies regarding ocular involvement in HLH.

Study	Year	Type	Age of patient(s)	Gender	Findings in patient(s) with HLH
Xu Li et al.^9^	2017	Case report	53 years old	Male	Unilateral panuveitis
Sebrow et al.^10^	2017	Case report	52 years old	Female	Purtscher retinopathy
Suhr et al.^11^	2016	Case report	1 month old	Female	Bilateral retinal white dot lesions which showed increased pigmentation over the next months.
Wang et al.^4^	2023	Retrospective analysis	133 patients with a mean age of 30.21 ± 14.42.	‐	Retinal/vitreous hemorrhage is the most common ocular finding in patients with HLH, followed by conjunctivitis, keratitis, anterior uveitis, and optic disc swelling.
Viscaino et al.^12^	2017	Case series	Three adult patients	Male	Pathologic evaluation revealed a bilateral uveal histiocytic infiltration with secondary involvement of the retina, optic nerve head, and trabecular meshwork.
Engelbert et al.^13^	2007	Case report	1 month old	Female	A one‐month‐old female with HLH, presented with bilateral posterior pole white dots predominantly involving the outer retina and choriocapillaris.
Chong et al.^14^	2012	Case report	Eight months old	Female	An eight‐month‐old girl with HLH presented with central nervous system involvement and optic nerve infiltration.

ONH involvement in HLH is rare. A wide range of differential diagnoses should be considered.^5^ We summarized the clinical approach to our patient in Figure 4. Regarding the patient's immune status, opportunistic infections such as CMV optic neuritis and retinitis must be investigated and ruled out. Ocular involvement in CMV infection is a serious sight‐threatening condition that could be manifested as ONH inflammation.^6^ Diagnosis of ocular CMV infection is clinical, although in atypical cases aqueous fluid PCR is diagnostic.^7^ The sensitivity and specificity of PCR tests for aqueous samples are 82% and 91%, respectively. The result of the aqueous and blood PCR tests was negative in our patient and we found no evidence of systemic CMV infection. In the brain and orbital MRI, we found bilateral optic nerve infiltration and the CSF analysis showed the brain involvement with tumoral cells. CNS involvement with infiltrative tumors can lead to increased ICP followed by bilateral ONH swelling. However, ICP was within normal limits. Another differential diagnosis in this patient is an adverse drug complication. There are reports of etoposide‐related optic nerve toxicity.^8^ However, clinical evidence and imaging findings indicate optic neuropathy due to tumoral infiltration, not drug toxicity.

## AUTHOR CONTRIBUTIONS

**Farid Shekarchian:** Investigation; supervision; writing – review and editing. **Mitra Karimi Amir Abadi:** Data curation; writing – original draft. **Mehrdad Motamed Shariati:** Investigation; supervision; writing – original draft; writing – review and editing.

## FUNDING INFORMATION

The authors received no funding. The author's work is not funded by the government or academic institutes.

## CONFLICT OF INTEREST STATEMENT

The authors declare that they have no competing interests.

## ETHICS STATEMENT

Not applicable.

## CONSENT

Written informed consent was obtained from the patient's legal guardian to publish this report in accordance with the journal's patient consent policy.

## Data Availability

The data that support the findings of this study are available on request from the corresponding author. The data are not publicly available due to privacy or ethical restrictions.
